# Longitudinal Sigma-Metric Profiling of Two Clinical Chemistry Platforms Using Peer-Group Internal Quality Control

**DOI:** 10.3390/diagnostics16162537

**Published:** 2026-08-12

**Authors:** Indah Meyliza, Aryati Aryati, Ferdy Royland Marpaung

**Affiliations:** 1 Clinical Pathology Subspecialization Program, Faculty of Medicine, Universitas Airlangga, Surabaya 60115, Indonesia; indahmeyliza@yahoo.com; 2 Department of Clinical Pathology, Faculty of Medicine, Universitas Airlangga, Surabaya 60115, Indonesia; aryati@fk.unair.ac.id; 3 Dr. Soetomo General Academic Hospital, Surabaya 60286, Indonesia

**Keywords:** six sigma metric, internal quality control, total allowable error, Standard Deviation Index, statistical quality control design, method comparison

## Abstract

**Background**: The Six Sigma metric benchmarks analytical performance, but most reports assess one platform at one time point, merging trueness with imprecision. Using routine peer-group internal quality control (IQC), we profiled two clinical chemistry platforms over five months. Because peer-group IQC compares imprecision directly but positions trueness only against same-method peers, we characterise rather than rank cross-platform trueness. **Methods**: Bio-Rad Unity peer-group IQC (January–May 2026) spanned two Siemens Dimension EXL analysers and two Roche cobas pro channels, two QC levels, and 30 analytes. TEa followed the CLIA 2024 Final Rule (25 analytes; provisional EFLM goals for five non-CLIA analytes). The Standard Deviation Index (SDI) referenced trueness to same-method peers, the CV ratio (CVR) imprecision, Sigma = (TEa − |bias|)/CV. Analyte-level comparison used Wilcoxon signed-rank testing, a confirmatory mixed-effects model, and Westgard SQC mapping. **Results**: Across 25 CLIA-regulated analytes, median Sigma was 5.9 (IQR 4.8–8.2) for cobas pro versus 4.0 (2.3–6.1) for Dimension EXL: a Hodges–Lehmann difference of 2.4 (95% CI 1.7–3.2; *p* < 0.001), confirmed by CLIA-only (2.51) and mixed-effects (2.7) analyses. Both aligned with their peers (SDI near zero); the gap reflected imprecision (median CV 1.7% vs. 2.5%; CVR 0.83 vs. 1.02), stable across months and instruments. The ranking reversed for iron, GGT and amylase; sodium, chloride, urea and creatinine were TEa-limited. The SQC redesign averaged N = 6 versus N = 3 controls/run (Dimension vs. cobas pro). **Conclusions**: A precision-driven difference separated the platforms as implemented here. Cross-platform trueness could not be ranked, yet decomposing Sigma localised precision limits, isolated calibration outliers, and produced analyte-specific SQC strategies needing prospective validation.

## 1. Introduction

Analytical quality underpins safe and comparable patient results, and the Six Sigma metric has become a standard way to express assay performance relative to the quality required for clinical use [1,2,3]. Written as σ = (TEa − |bias|)/CV, it combines trueness and imprecision against an allowable total error (TEa) goal and directly informs the selection of statistical quality control (SQC) rules and run sizes [4,5].

Despite its popularity, the published Six Sigma literature has four recurring limitations. Most reports are cross-sectional, capturing a single month or QC cycle and revealing little about the temporal stability of the estimate [2,6]. The majority also assess one platform in isolation, so the Sigma value carries no comparative reference and the analyte-specific drivers of poor performance remain obscured [7,8,9,10]. Sigma is almost always reported as a single composite number; because trueness and imprecision demand different corrective actions, collapsing them into one value obscures whether poor performance reflects a calibration or harmonisation problem or an inherent limit on precision [2,11,12,13]. Finally, benchmarking data from Southeast-Asian laboratories remain scarce, limiting the external validity of global quality benchmarks for the region [14,15,16].

We therefore carried out a longitudinal, dual-platform Sigma-metric profiling study in a high-volume tertiary academic laboratory, using routinely collected Bio-Rad Unity Real Time peer-group IQC data from five consecutive months. The design is layered: two platforms, two nominally identical instruments per platform, two QC levels, and five months of data. The analysis separates Sigma into a trueness component (the Standard Deviation Index, SDI) and an imprecision component (the CV ratio, CVR) [17]. Working within this framing, the study had three linked aims. The first was to test the between-platform Sigma difference and to establish whether it is driven by peer-relative trueness or by imprecision. The second was to identify analyte-level exceptions and local outliers that the decomposition can isolate, and to confirm within-platform inter-analyser concordance and temporal stability. The third was to translate the findings into an analyte-specific SQC plan that is offered as a recommendation requiring prospective validation.

## 2. Materials and Methods

### 2.1. Setting, Instruments, and Analytes

The study was conducted in the Department of Clinical Pathology of Dr. Soetomo General Academic Hospital and Faculty of Medicine, Universitas Airlangga (Surabaya, Indonesia), a tertiary referral and teaching laboratory. Two platforms were evaluated in parallel: two Siemens Dimension EXL analysers (DIM1 and DIM3, Siemens Healthineers, Malvern, PA, USA) and two Roche cobas pro channels (PROCC, configured as a cobas c 503 module, and PROCIM; Roche Diagnostics International Ltd., Rotkruez, Switzerland). Thirty routine clinical-chemistry analytes were measured at two internal quality control (IQC) levels on each system. IQC materials comprised Liquicheck Chemistry Control (Bio-Rad Laboratories, Inc., Irvine, CA, USA). The study used only de-identified aggregate QC statistics and involved no patient specimens or identifiable data.

### 2.2. Internal Quality Control and Data Extraction

Monthly (period, non-cumulative) IQC summaries from January to May 2026 were exported from the Bio-Rad Unity Real Time^®^ Online and Unity Web^®^ 2.0, version 1.1.59.8, Service Pack 59.8 (Bio-Rad Laboratories, Inc., Irvine, CA, USA). For every analyte–level–instrument–month combination we recorded the laboratory mean, standard deviation, coefficient of variation (CV), number of accepted QC points, and the corresponding peer-group statistics (mean, SD, CV, and number of contributing laboratories), together with the programme-reported SDI and CVR. Because Unity peer groups are method- and instrument-specific, each laboratory result was compared against laboratories using the same analytical method. Records with fewer than two accepted QC points, or with a non-evaluable CV, were excluded. Over the five-month window, routine reagent-lot replacement, QC-material lot changes, and scheduled preventive maintenance took place according to each analyser’s standard operating schedule. Because such events can shift bias and imprecision, and therefore Sigma, all analyte estimates were pooled across the full window rather than tied to a single lot or maintenance cycle, and lot- and maintenance-related variation is considered in the Limitations.

### 2.3. Allowable Total Error

The primary TEa goal was prespecified from the United States CLIA 2024 Final Rule for proficiency-testing acceptable performance (CMS-3355-F; effective 11 July 2024) [18]. Where an analyte is specified as the greater of a percentage or a fixed concentration limit (for example, creatinine ±10% or ±0.2 mg/dL), the effective percentage goal at each control level was calculated as max (percentage limit, absolute limit ÷ control mean × 100). Twenty-five of the thirty analytes are CLIA-regulated and form the primary analysis. The remaining five are not covered by CLIA (direct bilirubin, lipase, transferrin, lactate, and unsaturated iron-binding capacity); for these we applied provisional desirable TEa from the EFLM Biological Variation Database or, for UIBC, the CLIA total iron-binding-capacity limit as a surrogate, and flagged them accordingly [19,20]. These secondary goals are provisional, pending verification against the current EFLM database. We note that CLIA proficiency-testing limits are regulatory acceptability thresholds rather than biological-variation-derived specifications; for a minority of wide-goal analytes they yield very high Sigma values that are TEa-limited and should be interpreted as indicating ample analytical margin rather than as finely discriminating performance. The selection and use of allowable-error goals in a working laboratory, and the recognised strengths and limits of biological-variation-based specifications, follow current guidance [21,22]. Because Sigma depends directly on the TEa target chosen, the provenance of every goal is stated explicitly and interpreted with that dependence in mind [23].

### 2.4. Sigma Metric and Decomposition

For each analyte–level–instrument–month, bias was estimated against the peer group as (laboratory mean − peer mean) ÷ peer mean × 100, and Sigma was calculated as σ = (TEa% − |bias%|) ÷ CV% [2,7]. Performance was decomposed into two independent components reported directly by Unity. The SDI, defined as (laboratory mean − peer mean) ÷ peer SD, indexes trueness relative to peers; the CVR, defined as laboratory CV ÷ peer CV, indexes imprecision, with a value above 1 denoting worse-than-peer precision [17]. Bias and SDI are referenced to each method’s own peer group. They therefore express peer-relative alignment, not metrological trueness, and are not comparable across platforms, whereas the CVR is referenced in the same way but remains directly comparable. This split lets the SDI flag peer-relative calibration or harmonisation problems and the CVR expose inherent precision limits, and it isolates individual analysers whose local bias departs from an otherwise well-aligned peer group [12,13,24].

### 2.5. Statistical Analysis and SQC Redesign

Sigma distributions were summarised as the median (interquartile range) and as the proportion of QC events reaching σ ≥ 6 (world class), σ ≥ 4 (acceptable for routine SQC), and σ < 3 (unacceptable). Because the same analytes were measured on both platforms, the between-platform comparison was treated as paired at the analyte level rather than as independent QC events; the primary comparison used one Sigma per analyte–level per platform and the two-sided Wilcoxon signed-rank test, with the Hodges–Lehmann median difference and its 95% confidence interval as the effect size.

Of the 52 evaluable analyte–level pairs, 48 corresponded to the 25 CLIA-regulated analytes and the remaining 4 to two non-CLIA analytes (direct bilirubin, lipase) with EFLM-based provisional TEa that were fully evaluable on both platforms; these were included in the primary comparison to preserve statistical power, and a prespecified sensitivity analysis restricted to the 48 CLIA-regulated pairs is reported as the conservative reference (Section 3.1).

The single Sigma, CV, and bias values representing each analyte–level(–platform) combination—used in this comparison and in Table 1—were calculated as the median of the corresponding estimates across instruments and months, rather than by inserting pooled CV and bias into the Sigma formula. Because Sigma is a non-linear function of its inputs, the two approaches do not yield numerically identical values, and the median was chosen for robustness against months with very few accepted QC points.

A linear mixed-effects model with a random intercept for analyte, retaining the full event-level data, provided a confirmatory adjusted difference. Because Sigma is bounded and right-skewed, this model is a descriptive confirmatory adjunct rather than the primary inference, which rests on the distribution-free paired test.

Within-platform reproducibility was assessed by pairing the two instruments of each platform on common analyte–level combinations using the pooled five-month CV estimate rather than single-month values, which are unstable when monthly QC counts are low, and computing the mean absolute CV difference.

Temporal stability was examined by plotting monthly median Sigma for each instrument. Analyte- and level-specific median Sigma was mapped to a Westgard Sigma-metric SQC plan covering control rules, number of controls per run, and run frequency [4,5,25,26,27]. The probability of error detection and of false rejection of these plans was not prospectively simulated in this dataset. The plans are therefore presented as recommendations that require prospective validation before implementation rather than as validated control procedures.

**Figure 1:**
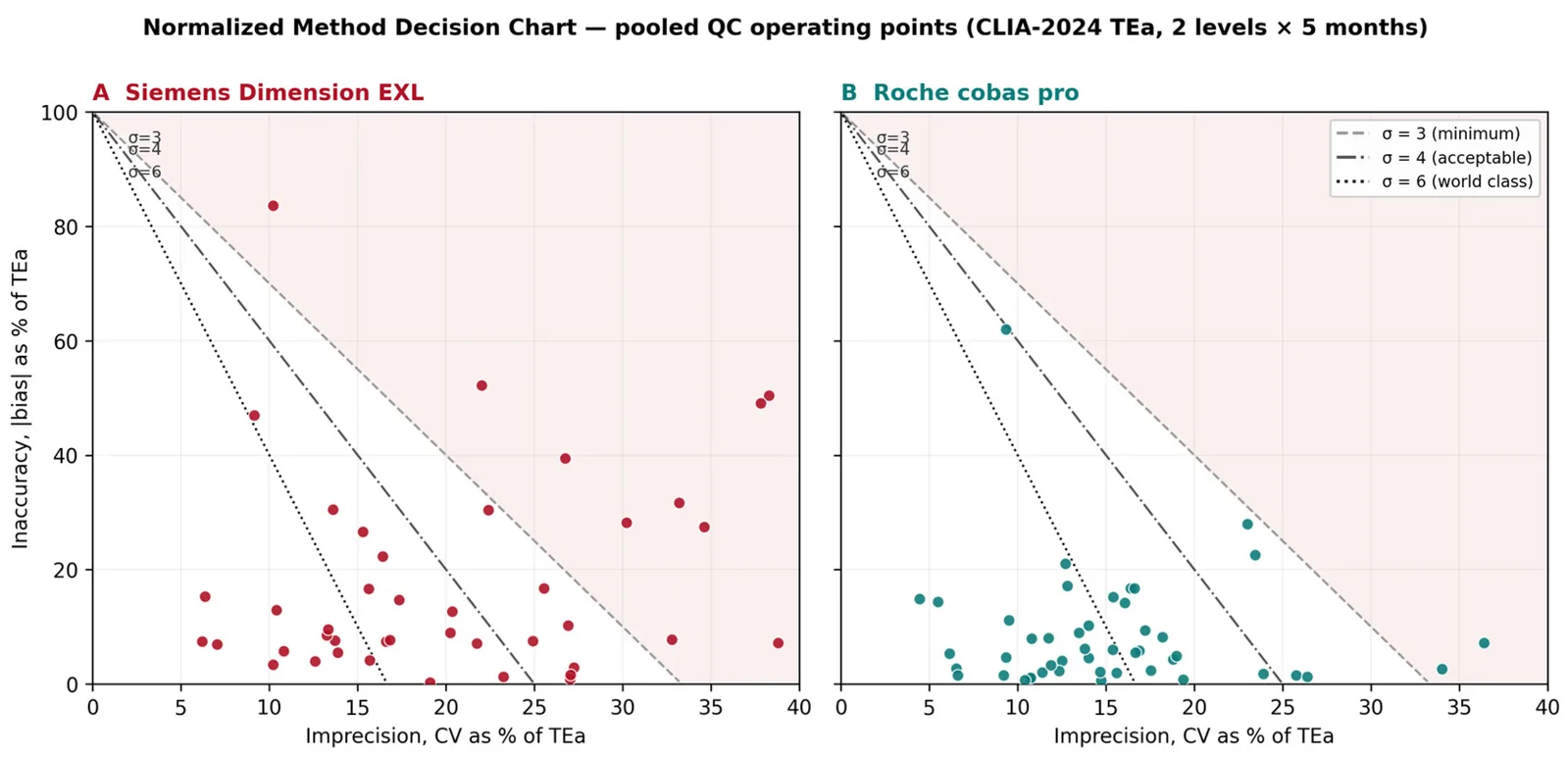
Normalised Method Decision Charts by platform. Each point is a pooled analyte–level operating point (CV ÷ TEa on the x-axis, |bias| ÷ TEa on the y-axis); diagonal lines are iso-Sigma decision boundaries and the shaded region marks σ < 3. The Siemens Dimension EXL (**A**) places many operating points in the rejection zone, whereas the Roche cobas pro (**B**) clusters in the high-Sigma region. TEa from CLIA 2024. Bias is peer-relative and method-specific (Section 2.4).

Performance was displayed on normalised Method Decision Charts (Figure 1), with each operating point plotted as imprecision (CV ÷ TEa) against inaccuracy (|bias| ÷ TEa) and iso-Sigma decision lines defined by |bias| + σ·CV = TEa [4,28,29]. Analyses were performed in R (version 4.4.1; R Foundation for Statistical Computing, Vienna, Austria) independently of the manufacturers; a two-sided α of 0.05 defined significance.

### 2.6. Data-Integrity Handling

Two integrity checks were prespecified. Lactate on the Dimension showed a peer mean (≈25, in mg/dL) roughly nine times the laboratory mean (≈2.9, in mmol/L), pointing to a unit and peer-group mismatch; these records were excluded from the Sigma analysis and are reported descriptively. The PROCIM channel was profiled in Unity under a cobas e 801 (immunoassay) label while running a clinical-chemistry menu, consistent with an instrument-profile mapping error; this did not affect the numerical QC statistics but is flagged for local correction. These two findings, uncovered during data curation, motivate the peer-group pitfalls discussed in Section 4 and prompted a systematic re-inspection of the remaining peer-group mappings, none of which showed comparable anomalies.

## 3. Results

After exclusions, 872 CLIA-regulated QC events across 25 analytes were available for the primary analysis, with a median of 17–18 accepted QC points per event on the Dimension and 46–48 on the cobas pro. This asymmetry in QC volume, considered further in the Discussion, reflects differing run and control frequencies on the two platforms.

### 3.1. Between-Platform Sigma Difference

Compared at the paired analyte–level unit (52 analyte–level combinations evaluable on both platforms, comprising 48 CLIA-regulated and 4 non-CLIA pairs; Section 2.5), and under this laboratory’s operating conditions, the Roche cobas pro showed a higher Sigma than the Siemens Dimension EXL: median Sigma was 5.9 (IQR 4.8–8.2) versus 4.0 (IQR 2.3–6.1), a Hodges–Lehmann median difference of 2.4 Sigma (95% CI 1.7–3.2; two-sided Wilcoxon signed-rank, *p* < 0.001), with the cobas pro superior in 46 of 52 pairs (88%). Because 4 of these pairs involved non-CLIA analytes with provisional TEa (direct bilirubin, lipase), the prespecified sensitivity analysis restricted to the 48 CLIA-regulated pairs yielded a highly consistent result (Hodges–Lehmann difference 2.51 Sigma, 95% CI 1.84–3.32; *p* = 1.3 × 10^−8^; cobas pro superior in 44 of 48 pairs, 92%), confirming that the difference was not an artefact of the TEa source. A linear mixed-effects model with a random intercept for analyte, retaining the full event-level dataset while accounting for repeated measurement of the same analytes, gave a concordant adjusted difference of 2.7 Sigma (95% CI 2.1–3.3). Because each platform comprised only two instruments, platform and instrument effects are not separately identifiable in a single model, so instrument-level performance is presented descriptively (Section 3.4). At the analyte–level unit, σ ≥ 6 was reached for 48% of cobas pro versus 27% of Dimension estimates, σ ≥ 4 for 80% versus 50%, and σ < 3 for 10% versus 33% (Figure 1).

### 3.2. Decomposition: Peer-Relative Trueness Versus Imprecision

**Figure 2:**
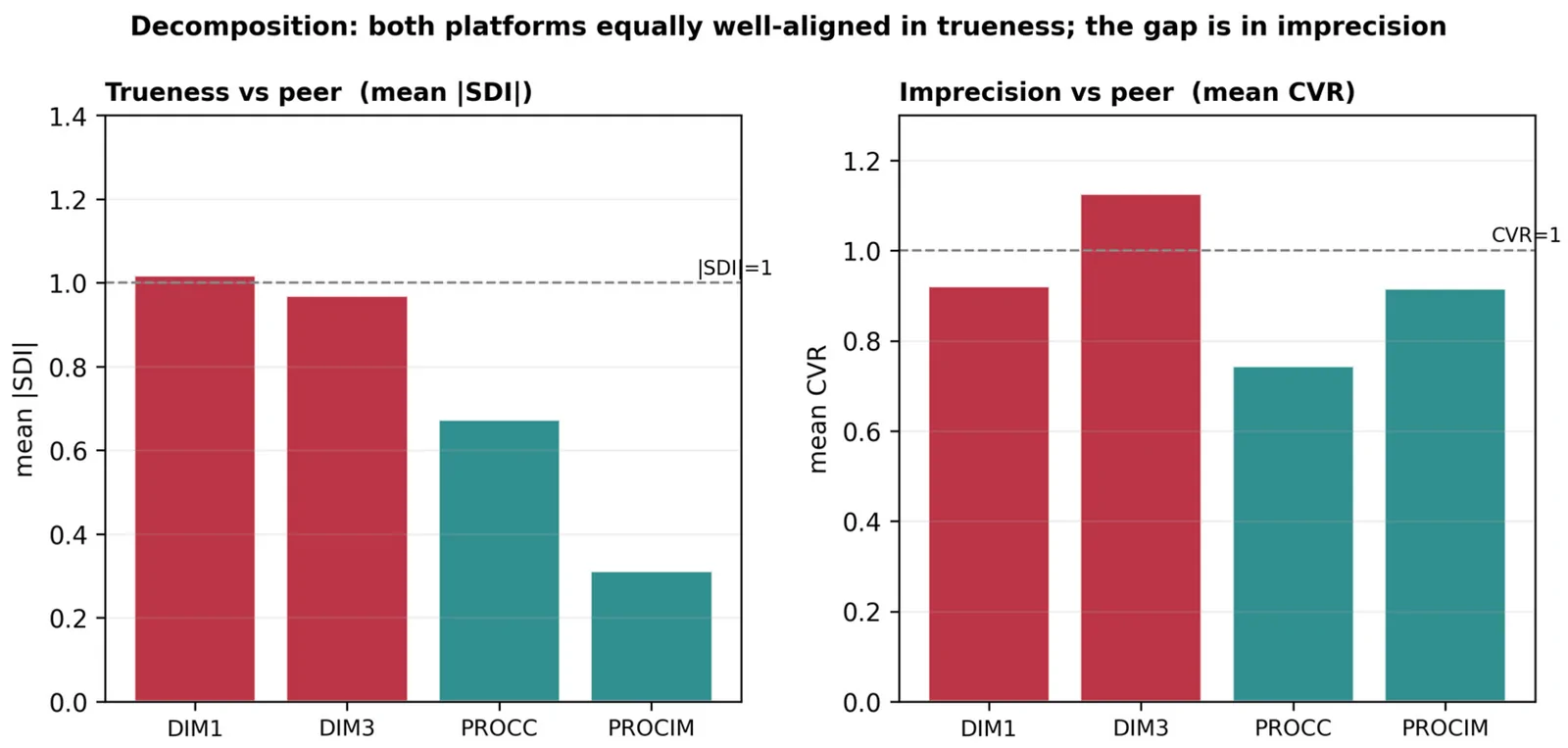
Decomposition of analytical performance. Mean |SDI| (peer-relative trueness, (**left**)) and mean CVR (imprecision, (**right**)) per analyser. Peer-relative alignment is comparable and near target across platforms; the measurable performance difference resides almost entirely in imprecision (CVR).

Each platform was well aligned with its own method-specific peer group. Mean SDI was close to zero on every system (DIM1 +0.01, DIM3 +0.06, PROCC −0.30, PROCIM −0.02), so neither platform carried a systematic offset against its peer consensus; this indicates good peer-relative alignment but does not establish equivalence of metrological trueness between platforms, which peer-group referencing cannot address. The measurable difference lay in imprecision: median laboratory CV was 1.7% for Roche versus 2.5% for Siemens, and mean CVR was 0.83 for Roche (better than peers) versus 1.02 for Siemens (on par with peers). The between-platform Sigma difference was therefore attributable to imprecision rather than to peer-relative harmonisation or calibration (Figure 2).

### 3.3. Analyte-Specific Performance

**Figure 3:**
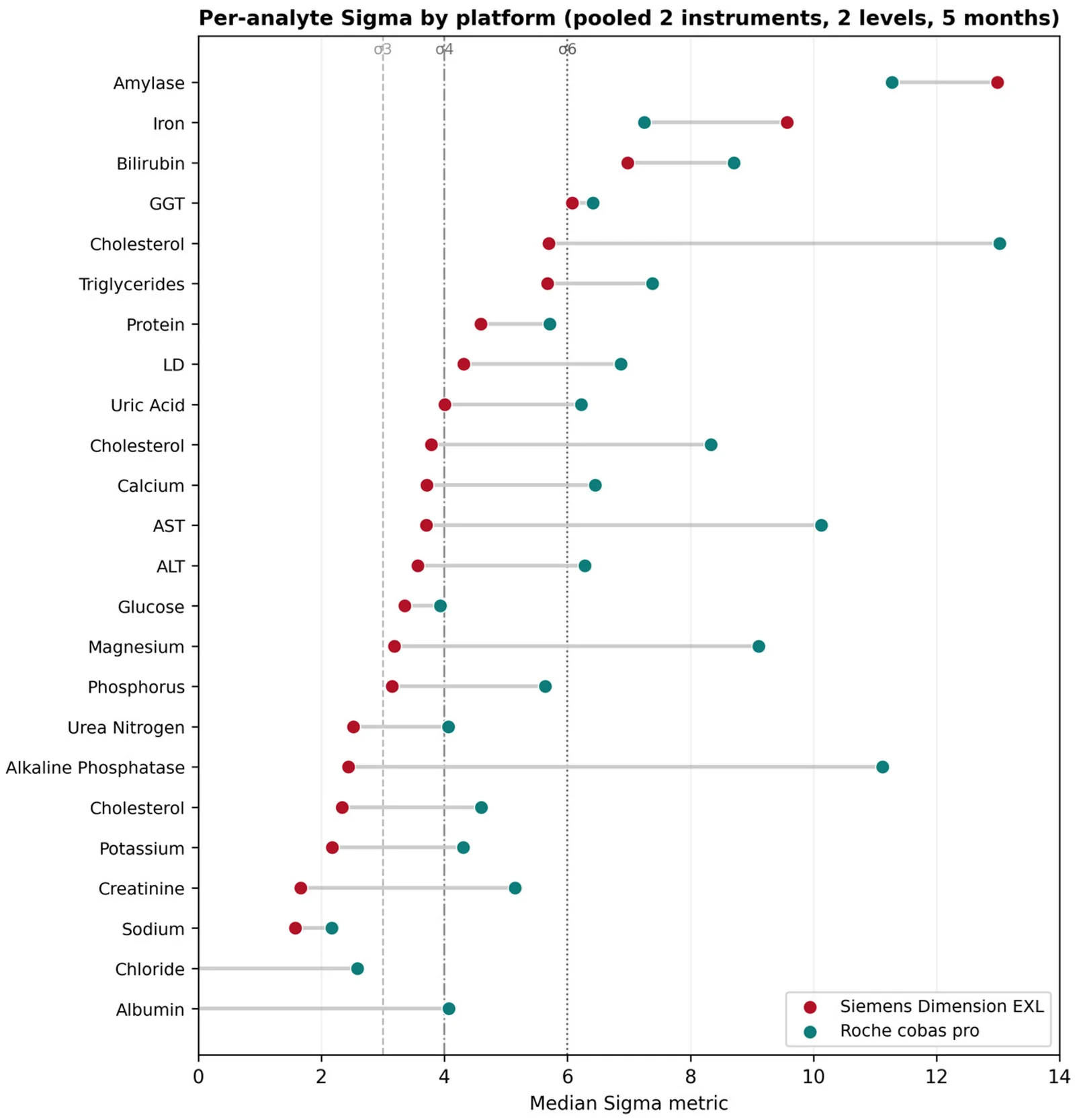
Per-analyte Sigma by platform. Median Sigma for each analyte, pooled across the two instruments, two levels, and five months. Connecting lines show the inter-platform gap. The vertical references mark the decision thresholds used in laboratory practice. A value of σ ≥ 6 denotes world-class performance that can be controlled with a single rule, σ ≥ 4 is acceptable for routine multirule control, σ = 3 is the minimum tolerable level, and σ < 3 is unacceptable and calls for method review rather than added controls. Inter-platform gaps that cross one of these thresholds, for example moving an analyte from below 4 to above 4, are the clinically actionable ones because they change the control strategy required.

The platform difference was strongly analyte-dependent rather than uniform (Table 1, Figure 3). Roche’s largest advantages were for alkaline phosphatase, aspartate aminotransferase, magnesium, HDL cholesterol, and LDL cholesterol. The Dimension, however, matched or exceeded the cobas pro for iron, gamma-glutamyl transferase, and amylase, which is inconsistent with a simple vendor effect and cautions against generalising the ranking to whole platforms. Analytes with intrinsically narrow goals—including sodium, chloride, urea nitrogen, and creatinine—gave the lowest Sigma values on both platforms, indicating that these are TEa-limited rather than artefactual. On the Dimension, 17 of 26 evaluable analytes fell below a median Sigma of 4, marking them as priorities for SQC redesign. Four analyte–level estimates returned a negative Sigma, all on the Dimension and all driven by bias exceeding TEa rather than by imprecision: albumin at both levels (bias up to +28% versus the method-specific peer group) and chloride at both levels. Because the reference here is the same-method peer consensus, a deviation of this magnitude points to a local calibration or reagent-lot problem on this specific analyser rather than to a generic method effect, and it is flagged for local calibration review; the decomposition isolates these peer-relative trueness failures from the platform-wide precision difference. The highest Sigma values (>12; e.g., CK, amylase, alkaline phosphatase) arose from very low CV (<1.5%) against wide allowable error and are TEa-limited (Section 2.3).

### 3.4. Within-Platform Inter-Analyser Concordance

When CV was estimated from pooled five-month data, the two nominally identical analysers of each platform were broadly concordant: the mean absolute difference in CV between DIM1 and DIM3 was 0.9% (Spearman r = 0.65 over 47 analyte–level pairs) with no systematic difference (Wilcoxon *p* = 0.75), and the two cobas modules differed by 0.6%. Their median Sigma values were closely matched (DIM1 3.5 vs. DIM3 3.6; PROCC 6.4 vs. PROCIM 6.7). The moderate rank correlation reflects the narrow spread of CV values across analytes rather than genuine disagreement, and the near-zero systematic difference is the more relevant metric for interchangeability. Apparent discordance emerged only when CV was compared month by month (mean |ΔCV| 2.7% for the Dimension), but this was an artefact of the small number of accepted QC points per month on that platform (median 18, several months below 10): with so few points the monthly CV estimate is itself unstable, and the difference expected between two such estimates from sampling variation alone (≈0.5%) accounts for most of the month-level signal. Inter-analyser comparisons based on peer-group CV should therefore be made on pooled rather than single-period data; on that basis the within-platform instruments are interchangeable, and the substantive comparability question is between the two platforms [1,30].

### 3.5. Temporal Stability

**Figure 4:**
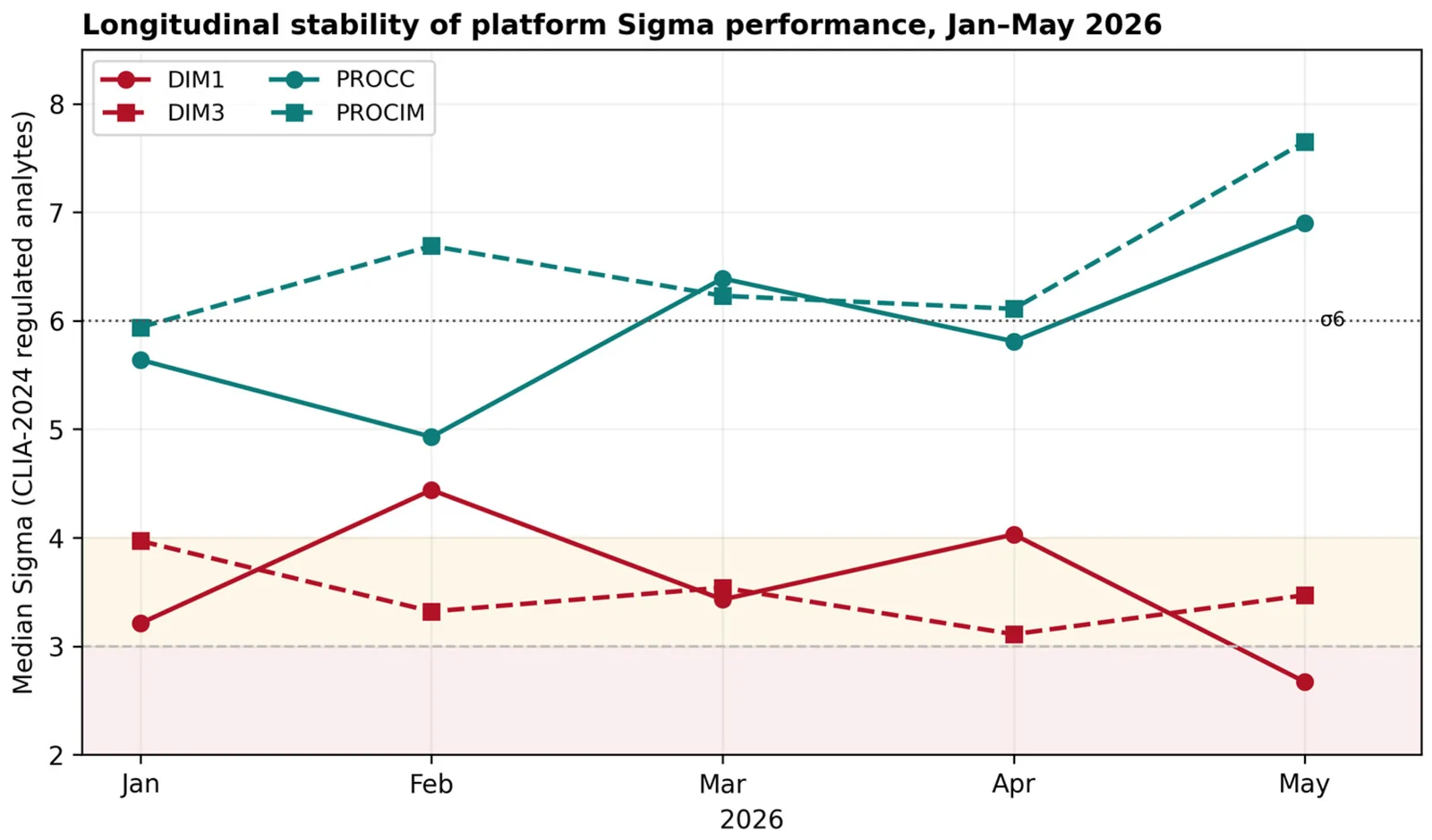
Longitudinal stability of platform Sigma, January–May 2026. Monthly median Sigma (CLIA-regulated analytes) for each analyser; shaded bands mark σ < 3 and σ 3–4. The Roche channels (teal) remain high and stable; the Dimension instruments (crimson) hover near σ = 3.

Performance was stable over the five months (Figure 4). The Roche channels remained in the high-Sigma region throughout and improved slightly towards May, while the Dimension instruments oscillated around a median Sigma of about 3 with no sustained improvement. The between-platform difference was therefore a persistent feature across the observation window rather than a transient single-month artefact, within the limits of a five-point series.

### 3.6. Sigma-Based SQC Redesign and Operational Cost

Mapping analyte- and level-specific Sigma to a Westgard SQC plan produced very different control burdens on the two platforms. High-Sigma cobas pro analytes could be run with a single rule and two controls per run. Low-Sigma Dimension analytes needed full multirule procedures with more controls, and sub-3-Sigma analytes needed root-cause investigation and method improvement before routine reporting. Averaged across analytes, the redesigned plan required about N = 6 controls per run on the Dimension against N = 3 on the cobas pro.

This burden tracks the Sigma distribution rather than the vendor label. On the Dimension, 15 of 24 evaluable analytes fell into the heaviest control category (full multirule, N ≥ 8) against 3 of 25 on the cobas pro, where 18 of 25 could be governed with N = 2. The few low-Sigma, TEa-limited cobas pro analytes (sodium, chloride) also required N ≥ 8. The practical message is that control effort should be set per analyte from its Sigma value rather than per platform. High-Sigma tests can be controlled more simply and less often, whereas sub-3-Sigma tests are not rescued by more controls and instead need method review. Because these selections come from published Sigma-to-rule tables rather than from prospective simulation, they are recommendations that require validation of their error-detection and false-rejection rates before local adoption [25,27]. The full per-analyte SQC plan is provided in the supplementary workbook.

## 4. Discussion

In this five-month, dual-platform study, and under the operating conditions of this laboratory, the Roche cobas pro showed a higher and statistically significant Sigma than the Siemens Dimension EXL, and that advantage was one of imprecision. Both platforms were well aligned with their own method-specific peer groups (SDI near zero), so the difference is not a peer-relative harmonisation problem and would not be corrected by recalibrating either system toward its peer consensus. It reflects the inherent precision of the systems as run in this laboratory, and a single composite Sigma would have masked this mechanism. As detailed in the Limitations, the design compares imprecision directly but does not rank cross-platform metrological trueness, and no such ranking is claimed.

The design combines elements rarely examined together. The longitudinal dimension separates a persistent performance difference from month-to-month noise. The paired-instrument dimension yields a methodological caution: the two nominally identical Dimension analysers appeared discordant when compared month by month, but this dissolved once CV was estimated on pooled data, where the instruments were concordant—apparent within-platform discordance was an artefact of small monthly QC counts rather than true instrument heterogeneity. The comparability question that remains is between the two platforms, whose different precision and method-specific calibration bear on result interchangeability when a patient is tested on one platform versus the other, as addressed by the comparability provisions of ISO 15189:2022 [1,30]. The analyte-level analysis cautions against over-generalisation: the platform ranking reverses for iron, GGT, and amylase, and several low-Sigma results (sodium, chloride, urea, creatinine) are TEa-limited rather than signs of poor methods [2,11].

The most direct application is the Sigma-driven SQC redesign. Beyond the established mapping of Sigma to control rules, our data quantify the downstream control burden: the lower-performing platform required roughly twice as many controls per run to be governed at equivalent Sigma-based rules, a figure laboratories can factor into procurement and consolidation decisions. For sub-3-Sigma analytes, no realistic SQC strategy can fully offset the analytical limitation, so the appropriate response is method improvement or reassignment rather than increasingly elaborate control rules [4,25]. Because these plans were derived from published Sigma-to-rule tables rather than validated prospectively, their error-detection performance should be confirmed by QC simulation before adoption.

The study also adds benchmarking evidence from a Southeast-Asian tertiary laboratory, a setting under-represented in the Six Sigma literature [7,14], and illustrates three peer-group QC pitfalls—a lactate unit mismatch, an instrument-profile mislabel, and the inflation of apparent inter-instrument discordance by unstable monthly CV estimates—that are easily overlooked yet can materially distort interpretation.

### Limitations

Several limitations qualify these findings. First, bias and trueness were assessed against Bio-Rad Unity peer-group consensus rather than certified reference materials or a commutable external-quality-assessment target; consensus is not metrological trueness, and a bias shared by all laboratories using a given method would go undetected. More fundamentally, Unity peer groups are method- and instrument-specific, so the SDI positions each platform relative to its own peer group and not relative to the other platform; the decomposition therefore demonstrates that each platform is well aligned with its own peer consensus but cannot rank cross-platform trueness. Second, each platform comprised only two instruments in a single centre, so platform and instrument effects are not separately identifiable and the findings describe these systems as implemented here rather than a generalisable vendor ranking. Third, the two platforms differed markedly in accepted QC points per event (median 17–18 vs. 46–48), which can affect the stability of monthly CV estimates; we mitigated this by basing precision comparisons on pooled five-month data, but residual influence cannot be excluded. Fourth, CLIA proficiency-testing limits are regulatory acceptability thresholds rather than biological-variation-based specifications and are permissive for several analytes, producing TEa-limited Sigma values above 10 that should not be over-interpreted; a biological-variation-based sensitivity analysis is a natural extension. Fifth, the longitudinal window comprised five monthly points, adequate to demonstrate persistence but limited for formal trend modelling. Finally, the SQC plans were mapped from published Sigma-to-rule tables and their probabilities of error detection and false rejection were not prospectively simulated. The redesigned rules were not implemented in routine operation during the study, so this work does not report whether they reduce false-rejection rates, improve error detection, or lower QC cost in practice. A prospective implementation study measuring these outcomes is the necessary next step. These constraints define the boundaries within which the conclusions hold.

## 5. Conclusions

Decomposing Sigma into its trueness and imprecision components showed that the two platforms were equally well aligned with their respective method-specific peer groups and that the measurable between-platform difference came from imprecision. Because peer-group referencing cannot rank metrological trueness across platforms, we characterise rather than adjudicate the difference. Reporting Sigma as its trueness and imprecision components, rather than as a single number, turns a benchmarking exercise into an analyte-specific tool for quality improvement and SQC design, and—together with the peer-group data pitfalls surfaced here—offers a transferable approach for laboratories running parallel platforms. The platform difference and the SQC redesign reported here describe these systems as implemented under the operating conditions of this laboratory and require prospective validation before they inform procurement or control decisions elsewhere.

## Figures and Tables

**Table 1:** Locked TEa goals and median Sigma per platform (pooled across instruments, levels, and months).

Analyte	TEa Source	TEa (%)	σ Siemens	σ Roche
ALT	CLIA 2024	15–23	3.6	6.3
AST	CLIA 2024	15	3.7	10.1
Albumin	CLIA 2024	8	−1.6	4.1
Alkaline phosphatase	CLIA 2024	20	2.4	11.1
Amylase	CLIA 2024	20	13	11.3
Bilirubin, total (TBIL)	CLIA 2024	20–42	7	8.7
CK	CLIA 2024	20	—	5.4
Calcium	CLIA 2024	8–12	3.7	6.5
Chloride	CLIA 2024	5	−1	2.6
Cholesterol, HDL	CLIA 2024	20–28	5.7	13
Cholesterol, LDL	CLIA 2024	20	3.8	8.3
Cholesterol, total	CLIA 2024	10	2.3	4.6
Creatinine	CLIA 2024	10–12	1.7	5.1
GGT	CLIA 2024	15	6.1	6.4
Glucose	CLIA 2024	8	3.4	3.9
Iron	CLIA 2024	15	9.6	7.2
LD	CLIA 2024	15	4.3	6.9
Magnesium	CLIA 2024	15	3.2	9.1
Phosphorus	CLIA 2024	10	3.1	5.6
Potassium	CLIA 2024	5–8	2.2	4.3
Protein, total, serum	CLIA 2024	8	4.6	5.7
Sodium	CLIA 2024	3	1.6	2.2
Triglycerides	CLIA 2024	15	5.7	7.4
Urea nitrogen	CLIA 2024	9–14	2.5	4.1
Uric acid	CLIA 2024	10	4	6.2
Bilirubin, direct (BC)	EFLM *	27	3.8	8.4
Lactic acid	EFLM *	30	—	15.2
Lipase	EFLM *	23	5.1	4.5
Transferrin	EFLM *	5	—	2.7
UIBC	TIBC–CLIA *	20	—	7.3

Cell shading: green σ ≥ 6, light-green σ ≥ 4, yellow σ 3–4, red σ < 3 (including negative). * Non-CLIA analytes use provisional EFLM or surrogate TEa pending verification; “—“ indicates the analyte was not evaluable on that platform. Negative Sigma denotes bias exceeding TEa (local calibration outlier; Section 3.3).

## Data Availability

De-identified aggregate IQC statistics, the full analytical dataset, per-analyte Sigma tables, longitudinal data, and the Sigma-based SQC plan are provided in the accompanying supplementary workbook and are available from the corresponding author on reasonable request.
